# Liquid Chromatography coupled with Mass Spectrometry as an Analytical Strategy to Assess the Occurrence of Potentially Toxic Cyanogenic Glycosides in Edible Microgreens

**DOI:** 10.3390/plants15091358

**Published:** 2026-04-29

**Authors:** Mariachiara Bianco, Ilario Losito, Beniamino Leoni, Onofrio Davide Palmitessa, Massimiliano Renna, Pietro Santamaria, Cosima Damiana Calvano, Tommaso R. I. Cataldi

**Affiliations:** 1 Dipartimento di Chimica, Università degli Studi di Bari Aldo Moro, Via E. Orabona 4, 70126 Bari, Italy; cosimadamiana.calvano@uniba.it (C.D.C.); tommaso.cataldi@uniba.it (T.R.I.C.); 2 Centro Interdipartimentale SMART, Università degli Studi di Bari Aldo Moro, Via E. Orabona 4, 70126 Bari, Italy; pietro.santamaria@uniba.it; 3 Dipartimento di Scienze del Suolo, della Pianta e degli Alimenti, Università degli Studi di Bari Aldo Moro, Via E. Orabona 4, 70126 Bari, Italy; beniamino.leoni@uniba.it (B.L.); onofrio.palmitessa@uniba.it (O.D.P.); massimiliano.renna@uniba.it (M.R.)

**Keywords:** cyanogenic glycosides, microgreens, flax, Brassicaceae, liquid chromatography, high-resolution mass spectrometry

## Abstract

Microgreens are increasingly promoted as sustainable, nutrient-dense foods, yet their content of potentially harmful specialized metabolites remains poorly explored. Here, we developed and applied a reversed-phase liquid chromatography–electrospray ionization high-resolution mass spectrometry (RPLC-ESI-HRMS) method for the detection of cyanogenic glycosides (CNGs) in edible microgreens. Method optimization, performed using dhurrin and lotaustralin as model standards, showed that positive ion detection of sodium adducts provided the most informative and selective HRMS/MS response, with diagnostic fragmentation patterns suitable for CNG recognition in complex matrices. Quantitative validation for lotaustralin showed excellent linearity (R^2^ = 0.998), low detection/quantification limits (LOD 0.16 mg/L; LOQ 0.53 mg/L), good extraction recovery, and a negligible matrix effect. Application of the method revealed a clear species-dependent profile. No detectable CNGs were found in broccoli raab and kale microgreens, supporting their safety as ready-to-eat products in this respect. In contrast, flax microgreens contained four CNGs: linamarin, lotaustralin, linustatin, and neolinustatin. Monoglycosylated species predominated, with lotaustralin quantified at 5.5 ± 0.6 mg/g dry weight and linamarin estimated at even higher levels. Diglycosylated CNGs were present at much lower concentrations and displayed multiple chromatographic peaks, consistent with the occurrence of structurally related isomeric forms. These quantitative results are specific to the flax microgreen samples analyzed here, obtained by pooling the lyophilized material obtained from several plants; thus, they do not account for biological variability among individual plants. Based on the measured CNG levels, flax microgreens showed a non-negligible cyanogenic potential. Assuming 1, 10 and 25% conversion to hydrogen cyanide, the estimated release would be, respectively, about 3, 33 and 81 mg HCN/kg of fresh flax microgreens, values lower than the current EU limit (150 mg HCN/kg of edible product) for flaxseed intended for direct consumption but comparable to values reported for other foods. These findings highlight the need to complement the nutritional evaluation of novel microgreens with targeted toxicological screening.

## 1. Introduction

Cyanogenic glycosides (CNGs) are naturally occurring plant compounds found in members of the *Fabaceae*, *Linaceae*, and *Rosaceae* families, including almonds and apricots, with particularly high concentrations reported in seeds and leaves [1]. Structurally, CNGs consist of a sugar moiety, most commonly glucose, linked through a glycosidic bond to an aglycone containing the cyanogenic group (CN) [2,3] (Figure S1 in the Supplementary Material). Their concentrations vary not only among plant species but also among different tissues within the same plant. In plant cells, CNGs are stored in vacuoles, spatially separated from endogenous hydrolytic enzymes such as β-glucosidases. When plant tissues are disrupted, enzymatic hydrolysis takes place, releasing HCN, a highly toxic compound that functions as a chemical defense against herbivores and pathogens [4].

In humans, the consumption of plants containing CNGs may cause side effects like nausea, vomiting, and diarrhea and, in severe cases, may result in death [5,6]. For this reason, plants with high CNG contents must be properly processed, for example, by cooking or other treatments, to reduce cyanide levels below a safe threshold [7]. Awareness of the presence of CNGs in certain foods, together with the application of appropriate preparation methods, is therefore essential to ensure consumer safety. Although CNGs are mainly recognized for their potential toxicity due to HCN release, some of them, such as amygdalin, have also been investigated for possible pharmacological properties, including anti-inflammatory and anticancer effects [8,9,10], but the latter remain controversial.

Among plant-derived foods that may pose a risk because of the occurrence of CNGs, innovative vegetables harvested at an early developmental stage have so far received limited attention. In recent years, microgreens have attracted growing interest because of their environmental sustainability, as their cultivation requires low water consumption, limited space, compatibility with vertical farming systems, and short harvesting cycles (approximately 20 days) [11]. Another distinctive feature of microgreens is that they are generally consumed raw, without cooking, unlike their mature counterparts. They are commonly used as garnishes or salad ingredients and are often appreciated for their high contents of minerals, vitamins, and antioxidants [11]. However, the possible negative effects on human health related to the presence of specific toxic metabolites in microgreens have not yet been adequately investigated. For this reason, edible microgreens from three plant species, previously analyzed in our laboratory for metabolites potentially beneficial to human health [12,13], were selected in the present study to evaluate the occurrence of CNGs. Broccoli raab and kale microgreens were selected among *Brassicaceae* species because the presence of glucosinolates, that is, compounds metabolically related to CNGs, had recently been demonstrated in these matrices [12]. Flax microgreens, previously found to contain interesting levels of potentially bioactive lipids [13], were chosen because flax seeds are well known to contain CNGs. European Regulation (EU) 2022/1364, issued in August 2022, establishes maximum permitted levels of hydrocyanic acid in certain food products, expressed as mg HCN per kg of food. For flax seeds intended for direct raw consumption, the maximum limit is 150 mg HCN kg^−1^ [14]. Regarding the relationship between CNGs and glucosinolates, it is worth noting that these two classes of compounds share common early biosynthetic steps; both derive from amino acids and involve aldoximes as key intermediates. Under specific conditions, plants that predominantly synthesize glucosinolates may therefore also produce CNGs [15,16].

In recent years, several studies have addressed the development of analytical methods for determining CNG levels in plant and biological matrices. Liquid chromatography (LC) with UV detection has been used to detect amygdalin, prunasin, linamarin, and related glycosides based on their characteristic absorption profiles, achieving satisfactory limits of detection [17]. Other studies have employed LC coupled with mass spectrometry (MS) because of its greater sensitivity and selectivity. Amygdalin was detected in its deprotonated form by MS with electrospray ionization (ESI) in negative ion mode and quantified by multiple reaction monitoring (MRM) using diagnostic fragment ions, such as those with *m*/*z* values of 323.0 and 178.7 [18,19]. In another study, linamarin and lotaustralin were quantified by RPLC-ESI(-)-MRM in 88 Trifolium species [20]. More recently, Zuk et al. [21] compared the contents of linamarin, lotaustralin, linustatin, and neolinustatin at different growth stages of flax plants, showing that monoglycosylated species were more abundant after approximately 7–21 days, whereas diglycosylated species predominated in seeds and in the first few days of growth (0–3 days) [21].

ESI in positive mode has also been widely used for the determination of CNGs in different matrices. Some studies took advantage of the strong affinity of the sugar moiety for sodium ions, promoting the formation of sodiated adducts, [M+Na]^+^, that were then quantified by MRM transitions based on diagnostic ions [22,23,24]. In other studies, the formation of ammonium adducts was promoted by adding ammonium formate to the chromatographic mobile phase, resulting in more pronounced fragmentation patterns [25,26,27]. Based on this background, the present work employed reversed-phase (RP) LC coupled with high-resolution/high-accuracy MS, using an ESI source operated in positive mode, for the identification of CNGs as sodium adducts in flax, kale, and broccoli raab microgreens.

## 2. Results and Discussion

### 2.1. Evaluation of RPLC-ESI-HRMS Conditions for CNGs Analysis

**Figure 1:**
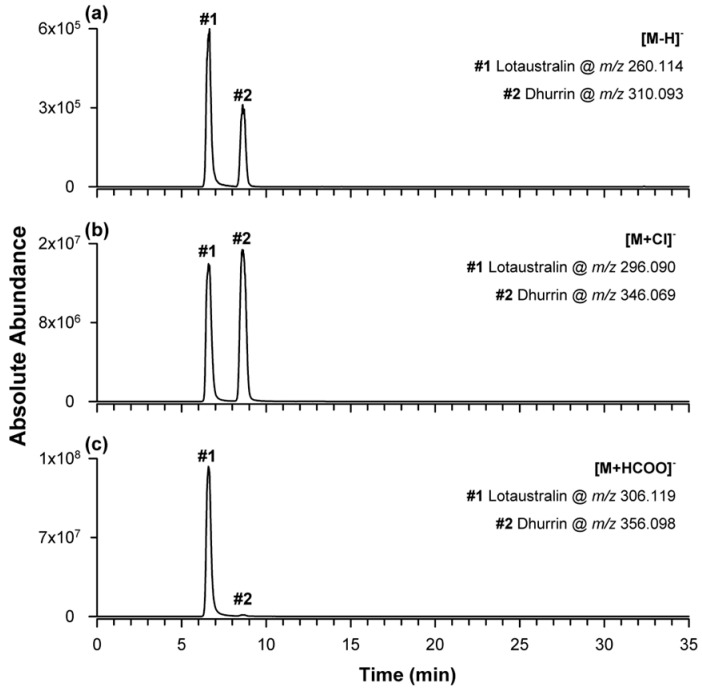
Extracted ion current (EIC) chromatograms of the ionic species detected for lotaustralin and dhurrin, both at 10 mg/L, by ESI in negative ion mode after RPLC separation using water and acetonitrile, both containing 0.1% formic acid, as mobile-phase solvents and the gradient elution program indicated as GE #1 in Section 3.4 and Figure S4. Deprotonated ions are shown in (**a**) at *m*/*z* 260.114 and 310.093, respectively; chloride adducts in (**b**) at *m*/*z* 296.090 and 346.069, respectively; and formate adducts in (**c**) at *m*/*z* 306.119 and 356.098, respectively.

Standard solutions of dhurrin and lotaustralin (10 mg/L) were analyzed using the chromatographic conditions described in Section 3, using ESI in either negative or positive ion mode. As shown in Figure S2, both compounds share the typical cyanogenic glycoside framework but differ in the aglycone substituents. Specifically, dhurrin contains a 4-hydroxyphenyl group and a hydrogen atom as R_1_ and R_2_ moieties, whereas lotaustralin bears methyl and ethyl groups, respectively. Negative ion ESI was first investigated after RPLC separation performed with a mobile phase of water and acetonitrile, both containing 0.1% formic acid. Under these conditions, three ionic species were detected for each analyte, namely the deprotonated molecule, the chloride adduct, and the formate adduct. Figure 1 shows the extracted ion current (EIC) chromatograms obtained by extracting the theoretical *m*/*z* values of the monoisotopic ions corresponding to dhurrin/lotaustralin deprotonated forms (Figure 1a), chloride adducts (Figure 1b), and formate adducts (Figure 1c), i.e., 310.093/260.114, 346.069/296.090, and 356.098/306.119, respectively.

Comparison of EIC peak intensities indicated that lotaustralin ionized most efficiently as the formate adduct (1.23 × 10^8^), whereas the deprotonated form and chloride adduct gave markedly lower responses (6.08 × 10^5^ and 1.31 × 10^7^, respectively). Dhurrin showed a different behavior: the chloride adduct gave the most intense signal (1.44 × 10^7^), followed by the formate adduct (1.11 × 10^6^) and the deprotonated ion (3.16 × 10^5^). This result was unexpected because chloride was not deliberately added to the mobile phase and was therefore attributable to solvent impurity, whereas formate ions originated from partial dissociation of formic acid in the mobile phase.

**Figure 2:**
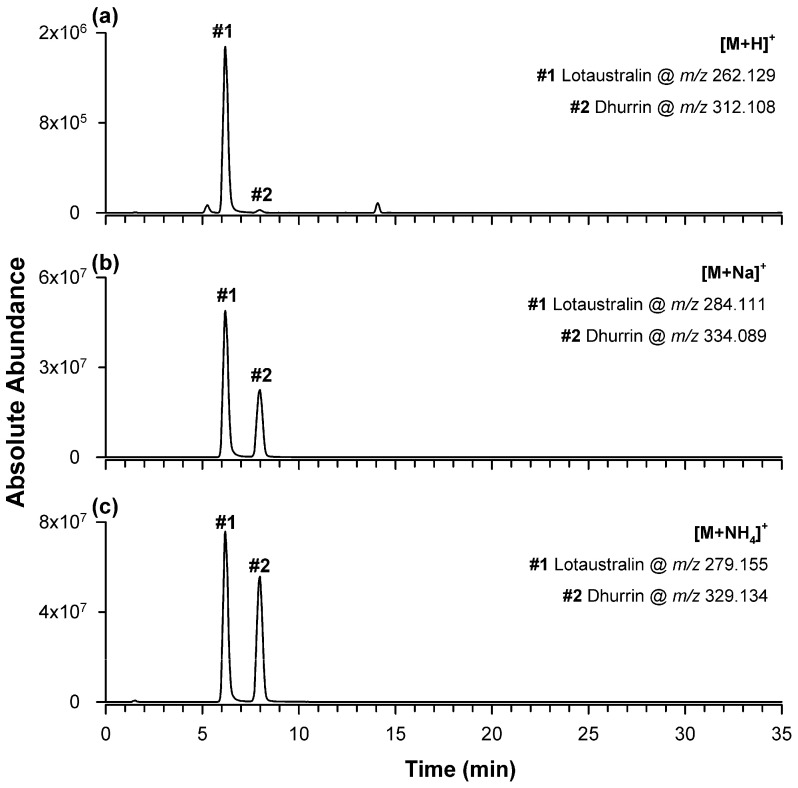
EIC chromatograms of the ionic species detected for lotaustralin and dhurrin, both at 10 mg/L, by ESI-MS in positive ion mode after RPLC separation using water and acetonitrile containing 0.1% formic acid, with 5% water containing 20 mM ammonium formate in the mobile phase and GE #1. Protonated ions are shown in (**a**) at *m*/*z* 262.129 and 312.108, respectively; sodium adducts in (**b**) at *m*/*z* 284.111 and 334.089, respectively; and ammonium adducts in (**c**) at *m*/*z* 279.155 and 329.134, respectively.

To assess whether negative ionization could be driven toward the formation of a single ionic species, ideally the formate adducts, and thus improve method sensitivity, a second chromatographic separation procedure was carried out using water containing 20 mM ammonium formate as a third solvent, kept at a fixed 5% value throughout the gradient. However, analysis of the dhurrin/lotaustralin mixture under these conditions did not produce substantial changes in the relative abundance of negatively charged species compared with the previous experiment. Positive ion ESI-MS was then evaluated under the same chromatographic conditions. The corresponding EIC traces are shown in Figure 2.

In this case, peaks were observed for the exact *m*/*z* values of protonated molecules (Figure 2a), sodium adducts (Figure 2b), and ammonium adducts (Figure 2c) of the two CNGs. Both analytes exhibited comparable responses for sodium (4.89 × 10^7^ for lotaustralin and 2.24 × 10^7^ for dhurrin) and ammonium adducts (7.11 × 10^7^ for lotaustralin and 5.21 × 10^7^ for dhurrin), whereas protonated species were much less abundant, particularly for dhurrin, whose protonated ion was barely detectable (1.39 × 10^6^ for lotaustralin and 2.39 × 10^4^ for dhurrin) (Figure 2a). Thus, also in positive mode, no single ionic species overwhelmingly prevailed. Nevertheless, sodium adducts were selected as target ions for further experiments because they provided more reproducible chromatographic responses and, most importantly, more informative and diagnostically useful HRMS/MS spectra than the corresponding ammonium adducts. A notable result was the high abundance of sodium adducts, despite the absence of intentional sodium ion addition. This behavior, observed even in the presence of a much higher concentration of ammonium ions, suggests a pronounced affinity of both analytes for sodium, plausibly due to coordination involving the hexose moiety. This process is consistent with the well-known affinity of carbohydrate-containing molecules for alkali metal ions in electrospray ionization conditions [28]. Addition of 2 mM sodium acetate to solvent A further promoted sodium adduct formation, but also introduced significant chromatographic noise, making the approach unsuitable for real samples.

**Figure 3:**
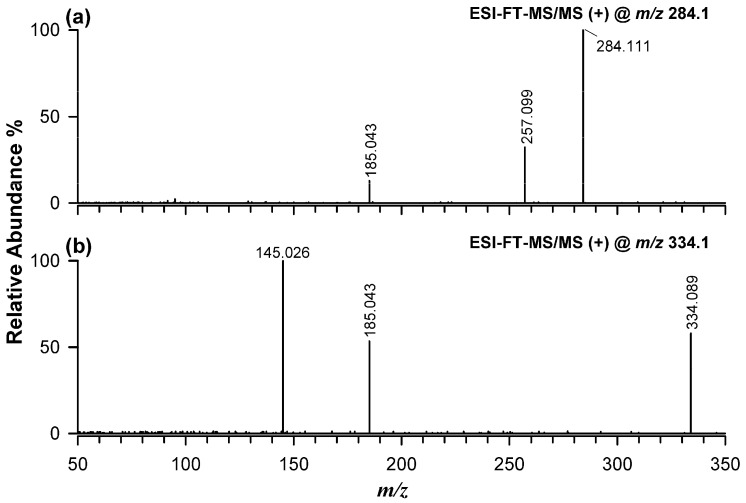
ESI(+)-HRMS/MS spectra of the sodium adducts of lotaustralin at *m*/*z* 284.1 (**a**) and dhurrin at *m*/*z* 334.1 (**b**).

Based on these results, RPLC-ESI-HRMS analysis performed in positive ion mode with water and acetonitrile, both containing 0.1% formic acid, was selected for further work. Under these conditions, both dhurrin and lotaustralin were detected mainly as sodium and ammonium adducts, with sodium adducts prevailing. Moreover, RPLC-ESI(+)-HRMS/MS experiments demonstrated that sodium adducts generated more informative tandem mass spectra than ammonium adducts. Figure 3 reports the MS/MS spectra of the sodium adducts of lotaustralin at *m*/*z* 284.111 (Figure 3a) and dhurrin at *m*/*z* 334.089 (Figure 3b).

In both cases, precursor ion mass errors were within acceptable limits for high-accuracy measurements, supporting confident elemental composition assignment. For lotaustralin, a fragment ion at *m*/*z* 257.099 was observed, corresponding to loss of HCN and potentially leading to two alternative product ions, as shown in Figure S2. A less intense signal at *m*/*z* 185.043 was assigned to the sodium adduct of a dehydrated sugar moiety (monoisotopic mass at *m*/*z* 185.042), generated through loss of methyl, ethyl-cyanohydrin (Figure S2). The same ion was also detected in the MS/MS spectrum of the dhurrin sodium adduct, consistent with neutral loss of hydroxymethyl-cyanohydrin (Figure S2). The dominant fragment in the dhurrin spectrum appeared at *m*/*z* 145.026 and was assigned to the sodium adduct of 4-hydroxybenzaldehyde. As shown in Figure S2, the formation of this ion likely involves the loss of a dehydrated glucose molecule, rapidly followed by HCN loss, since the intermediate product ion (exact *m*/*z* 172.037, see Figure S2) could not be detected in the MS/MS spectrum of the dhurrin sodium adduct (see Figure 3b).

These characteristic product ions indicate that MS/MS spectra of sodium adducts can support recognition of CNGs in real samples, even in the presence of co-extracted isobaric or isomeric metabolites. The combined use of accurate mass, retention behavior, and diagnostic fragmentation therefore provided a robust basis for the subsequent annotation of CNGs in microgreen extracts. Accordingly, RPLC performed with water and acetonitrile containing 0.1% HCOOH, followed by positive ion ESI-MS detection, was selected for method validation and for subsequent identification and quantification of cyanogenic glycosides in microgreen extracts.

### 2.2. Identification of CNGs in Microgreen Samples

**Figure 4:**
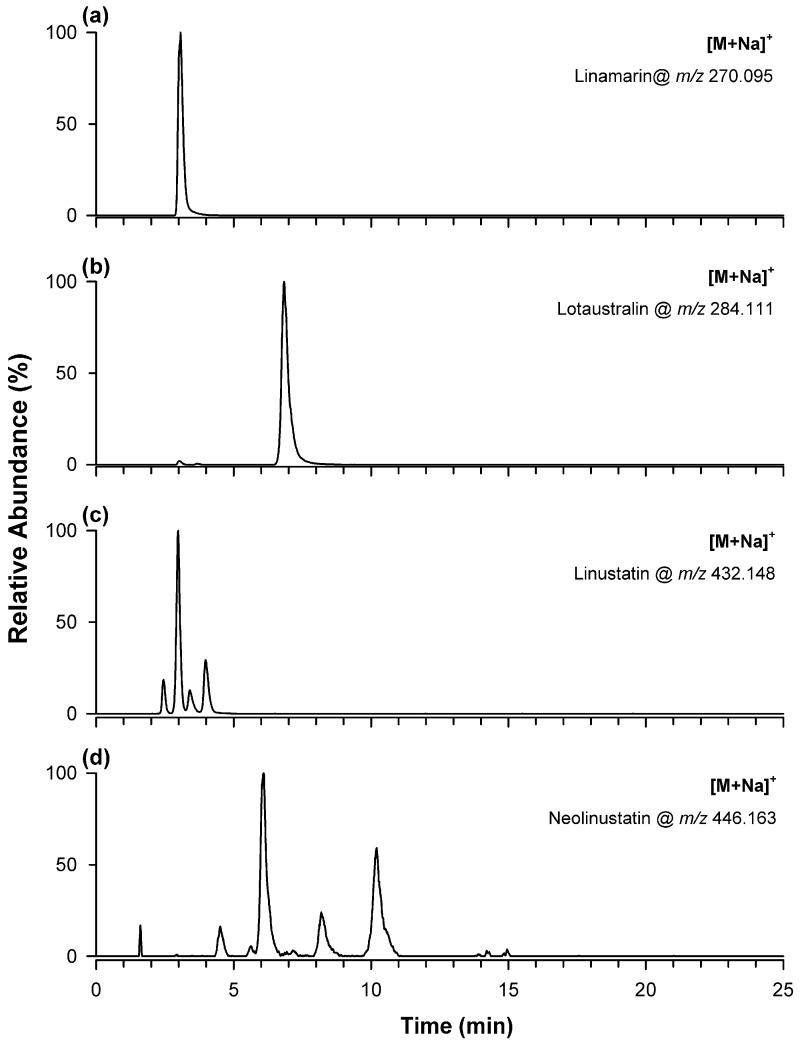
EIC chromatograms of the sodium adducts of cyanogenic glycosides detected in one extract of flax microgreens by RPLC-ESI-HRMS, with GE #1: linamarin at *m*/*z* 270.095 (**a**), lotaustralin at *m*/*z* 284.111 (**b**), linustatin at *m*/*z* 432.148 (**c**), and neolinustatin at *m*/*z* 446.163 (**d**). The presence of multiple peaks in (**c**,**d**) may indicate the coexistence of structurally related isomeric forms, possibly arising from differences in hexose composition and/or conformational variability of the glycosidic residues. Lotaustralin concentration in the specific sample was 5.5 ± 0.6 mg/g DW. Linamarin, linustatin and neolinustatin concentrations were estimated, respectively, as 24 ± 3, 0.79 ± 0.07, and 0.0897 ± 0.0014 mg/g DW.

RPLC-ESI(+)-HRMS analysis of flax microgreen extracts enabled the tentative identification of four cyanogenic glycosides, namely linamarin, lotaustralin, linustatin, and neolinustatin, based on accurate mass, expected sodium adduct formation, chromatographic behavior, and diagnostic HRMS/MS fragmentation (see Table S1). As shown in Figure S3, linamarin and lotaustralin are monoglycosylated compounds, differing only for the presence of a methyl group in linamarin and an ethyl group in lotaustralin. Linustatin and neolinustatin are the corresponding diglycosylated derivatives. Figure 4 reports the EIC chromatograms obtained for the sodium adducts of linamarin at *m*/*z* 270.095 (Figure 4a), lotaustralin at *m*/*z* 284.111 (Figure 4b), linustatin at *m*/*z* 432.148 (Figure 4c), and neolinustatin at *m*/*z* 446.163 (Figure 4d) in a flax microgreen extract. A single chromatographic peak was observed for each monoglycosylated species, with lotaustralin eluting after linamarin (6.8 vs. 3.1 min), consistently with the higher hydrophobicity of the ethyl group relative to the methyl group. In contrast, the diglycosylated species exhibited more complex chromatographic profiles, suggesting the presence of multiple structurally related forms. Linustatin gave peaks at 2.5, 3.0, 3.4, and 4.0 min, whereas neolinustatin produced peaks at 4.5, 6.1, 8.2, and 10.2 min, together with two weak additional peaks close to 6.1 min and a clear shoulder on the right side of the last peak.

It should be emphasized that, although the CNGs discussed above eluted within the first 15 min, the chromatographic run was intentionally extended beyond this time window by incorporating a gradient step with a slow increase in acetonitrile content (see GE #1 in Figure S4). In particular, the shallow increase from 15% to 18% acetonitrile was specifically designed to improve the separation of additional cyanogenic glycosides that might be present in the extracts and that, owing to their more hydrophobic moieties, would be expected to elute later than the four compounds described above. This precaution was particularly relevant for broccoli raab and kale microgreens, whose extracts were considered more likely to contain such compounds (*vide infra*). More generally, the intermediate steps with smaller increases in acetonitrile, preceding the final increase to 95% of this solvent, also aimed to achieve smoother elution of co-extracted matrix constituents that are more hydrophobic than the detected CNGs. This is supported by the TIC chromatogram of flax microgreen extract obtained by RPLC-ESI-HRMS (Figure S4), in which several convolved peaks appear after the 2–15 min retention interval where the CNGs detected in the same sample eluted. This chromatographic feature indicates the presence of numerous matrix components more hydrophobic than the analytes of interest.

Notably, some of these components eluted only after 30 min, i.e., during the isocratic step at 95% acetonitrile, when the mobile phase had its maximum elution strength. Furthermore, the final 14 min re-equilibration step at 5% acetonitrile was retained to ensure adequate column reconditioning and to improve method robustness during the analysis of the complex plant extracts investigated here. A similar chromatographic pattern was observed also for broccoli raab and kale microgreens. Notably, Figure S4 also compares the GE program considered in this study (GE #1, blue trace) with a shorter 37 min method (GE #2, red trace). The latter, involving a shallow increase in acetonitrile percentage only from 15 to 16.5% between 8 and 15 min (to reproduce the same slope as GE#1 in the corresponding step) and then a sharp increase to the final 95% value, may be suitable for the separation of CNGs detected in flax microgreens (all eluting within 15 min), but would lead to the very fast elution of more hydrophobic matrix constituents, with potential risks of carry-over and/or problems in separation reproducibility; thus, it might be more appropriate for samples with a lower content of such compounds.

**Figure 5:**
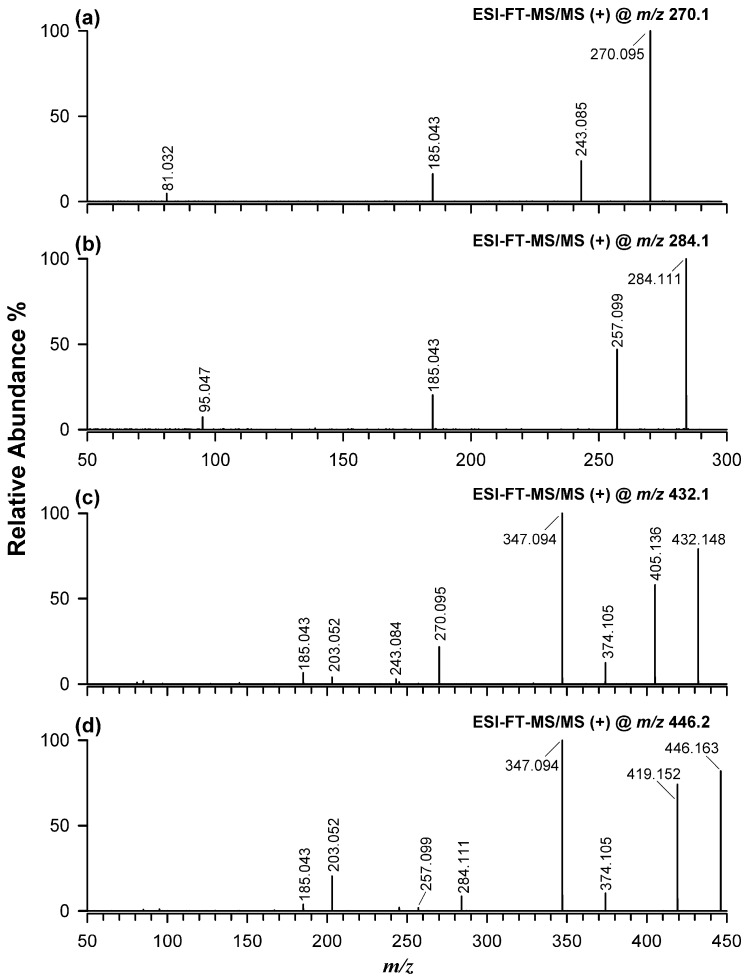
ESI(+)-HRMS/MS spectra of the sodium adducts of cyanogenic glycosides detected in flax microgreens: linamarin at *m*/*z* 270.1 (**a**), lotaustralin at *m*/*z* 284.1 (**b**), linustatin at *m*/*z* 432.1 (**c**), and neolinustatin at *m*/*z* 446.2 (**d**). For linustatin and neolinustatin, the reported spectra were averaged over the major peaks observed in the corresponding EIC chromatograms (see Figure 4).

Structural confirmation for CNGs detected in flax microgreens was obtained by means of tandem MS with a different identification level for each CNG, as reported in Table S1 [29]. Figure 5 reports the MS/MS spectra averaged under the chromatographic peaks of linamarin (Figure 5a) and lotaustralin (Figure 5b), and under the major peaks among those detected for linustatin (Figure 5c) and neolinustatin (Figure 5d). Importantly, all peaks assigned to linustatin and neolinustatin showed the same product ions, although relative intensities varied slightly. In the absence of authentic standards for these diglycosylated compounds, the corresponding chromatographic peaks were interpreted as related to putative isomeric and/or conformational forms.

The sodium adducts of the monoglycosylated species displayed closely similar fragmentation patterns, including the characteristic loss of HCN, yielding ions at *m*/*z* 243.085 and 257.099 for linamarin and lotaustralin, respectively, together with the diagnostic fragment at *m*/*z* 185.043. Minor ions at *m*/*z* 81.032 and 95.047 were also observed and, as shown in Figure S3, can be assigned to aglycone-derived sodium adducts generated after the loss of dehydrated glucose and further HCN elimination.

As expected, the MS/MS spectra of the diglycosylated species (Figure 5, plots c and d) were more complex, but still revealed several common fragmentation pathways, starting with the expected neutral loss of HCN and formation of ions at *m*/*z* 405.136 and 419.152 for linustatin and neolinustatin, respectively. Additional common fragment ions at *m*/*z* 374.105 and 347.094 were consistent with a fragmentation route analogous to that previously described for the dhurrin sodium adduct, namely release of a CN-modified sugar-containing moiety (see Figure S2). In the case of the diglycosylated compounds, the ion at *m*/*z* 374.105 can be rationalized by 1,3-transfer of the CN group to the disaccharide moiety, followed by release of the corresponding sodium adduct and neutral loss of acetone or methyl-ethyl-ketone, depending on the compound, whereas the ion at *m*/*z* 347.094 arises from subsequent loss of HCN from the CN-modified disaccharide (see Figure S5). As shown in the figure, ions at *m*/*z* 270.095 and 284.111, observed in the MS/MS spectra of linustatin and neolinustatin, respectively, correspond to the sodium adducts of the related monoglycosylated species formed after loss of dehydrated glucose. These ions were accompanied by product ions at *m*/*z* 243.084 and 257.099, respectively, generated by further loss of HCN. Finally, common ions at *m*/*z* 203.052 and 185.043 were assigned to the sodium adduct of glucose (exact *m*/*z* 203.053) and its dehydrated form (exact *m*/*z* 185.042), respectively (Figure S5).

Overall, these results confirm that the MS/MS spectra of sodium adducts provide valuable diagnostic information for distinguishing CNGs, including diglycosylated species, from isobaric/isomeric co-extracted metabolites. However, the fragmentation spectra of the multiple diglycosylated species separated by RPLC were qualitatively identical. This behavior may reflect the presence of isomeric forms differing in hexose identity and/or molecular conformation, as previously hypothesized by Yulvianti and Zidorn [2] for plant CNGs. Such structural variability is expected to influence interaction with the C18 stationary phase, thereby causing differences in retention time [19,30], while leaving the overall MS/MS fragmentation pattern essentially unchanged. Accordingly, retention-time differences alone were not considered sufficient for unambiguous structural discrimination among isomeric forms. In this case, only variations in relative fragment intensities would be expected, reflecting different fragmentation efficiencies among conformers, as already observed by MALDI-MS [31].

RPLC-ESI(+)-HRMS analyses were also performed on broccoli raab and kale microgreens to investigate the possible occurrence of CNGs. Indeed, a recent study carried out in our laboratory demonstrated that these Brassicaceae microgreens contain several glucosinolates [12], i.e., metabolites derived from a parallel biosynthetic pathway [15,16]. Accordingly, the occurrence of CNGs bearing R groups analogous to those of the glucosinolates identified in the same matrices was explored by extracting the ion currents corresponding to their sodium adducts from the RPLC-ESI(+)-HRMS data. Within the sensitivity limits of the present method, the lack of a significant response, even though the RPLC elution program was purposely designed to favor the separation of further CNGs, more hydrophobic than those detected in flax microgreens, indicates that these compounds, if present, occur at concentrations below the detection capability achieved for these matrices. It is worth noting that cyanohydrins related to glucosinolates previously detected in the microgreens of the two *Brassica* plants were also searched for in their extracts using RPLC-ESI-HRMS, since a metabolic pathway generating them from glucosinolates, instead of CNGs, has been reported [16]. However, they could not be detected, thus suggesting that the possible generation of cyanohydrins by glucosinolates, in the absence of CNGs, can be considered negligible in those products.

These observations further support the safety of kale and broccoli raab microgreens as ready-to-eat products for human consumption. By contrast, because CNGs were clearly detected in flax microgreens, the analytical method was subsequently validated for a quantitative application to this matrix, starting from lotaustralin as a target analyte.

### 2.3. Validation and Application of the RPLC-ESI(+)-HRMS Method to the Determination of Lotaustralin and Related CNGs in Flax Microgreens

Calibration for lotaustralin was first performed using standard solutions in pure solvent, with the EIC peak area of the sodium adduct adopted as the analytical response. As shown in Figure S6, excellent linearity was obtained over the concentration range 0.5–10 mg/L (with three replicates performed at each level). LOD and LOQ values of 0.16 and 0.53 mg/L, respectively, were obtained. The within-day and between-day precision of analytical responses was subsequently assessed, considering RSD values for EIC peak areas obtained for sets of three replicated analysis of lotaustralin at a 0.5 mg/L concentration, each performed in three different days during the same week. Despite the low analyte concentration considered for the test, within-day RSD values did not exceed 4%, whereas the between-day RSD was 6%, thus confirming the excellent reproducibility of the analytical response over a reasonable time range.

Matrix effects were then evaluated in flax microgreen samples, which naturally contain lotaustralin. In detail, a matrix-matched calibration (see Figure S5) was obtained considering aliquots of flax microgreens extract spiked with standard lotaustralin within the same concentration range adopted for the calibration in pure solvent (0.5–10 mg/L). The matrix effect was then assessed by calculating the percentage ratio between the slope of the calibration line obtained for the spiked samples and that of the calibration line resulting from solutions in pure solvent. The obtained value (104%) indicated the absence of a significant matrix effect, most likely because of sample dilution prior to LC-MS analysis.

Extraction recovery was assessed by performing four consecutive extraction cycles, each consisting of two extraction steps, on the same aliquot of lyophilized flax microgreens, as described in the Section 3, taking the cumulative peak area obtained across all extractions as 100% recovery. The experiment was carried out in triplicate using different aliquots from the same batch. Recoveries were 87± 6% for the first cycle, 11.4 ± 0.6% for the second, 1.57 ± 0.13% for the third, and 0.087 ± 0.009% for the fourth. Each value was calculated as the ratio between the response obtained in each cycle and the sum of the responses obtained over the four cycles, assuming that no further analyte could be recovered thereafter. This assumption appears reasonable in view of the sharp decrease in response observed from one cycle to the next. These data indicate that the first extraction cycle provides good recovery of lotaustralin and that the extraction procedure is reproducible.

Lotaustralin was then quantified in flax microgreens by analyzing three independent aliquots of lyophilized material and correcting the measured values for both recovery and matrix effect. The resulting concentration was 5.5 ± 0.6 mg/g dry weight (DW). Since dry weight accounted for approximately 10% of fresh weight in the analyzed product, this value corresponds to 0.55 ± 0.06 mg/g fresh weight (FW). The concentration found in the present study was lower than that reported by Zuk et al. [21] for flax plants harvested at comparable developmental stages, as those authors found 10–20 mg/g DW in samples collected 7–14 days after sowing. This difference may reflect variation in genotype, cultivation conditions, or harvest stage, which are known to affect cyanogenic glycoside accumulation.

After lotaustralin quantification, the levels of the other detected CNGs were semi-quantified from their EIC peak areas (see Table S1). Because authentic standards were not available, comparable ionization efficiency as sodium adducts was assumed based on the close structural similarity of these compounds to lotaustralin. The resulting quantitative data should therefore be regarded as approximate estimates. Moreover, their estimated concentrations were all above the thresholds established for lotaustralin, and all corresponding EIC peaks showed signal-to-noise ratios higher than 10.

Using this approach, linamarin was semi-quantified at 24 ± 3 mg/g DW, thus occurring at a substantially higher level compared to lotaustralin. This result is qualitatively consistent with the data reported by Zuk et al. [21], who found higher concentrations of linamarin than lotaustralin in flax harvested 7–14 days after sowing (25–30 vs. 10–20 mg/g DW), although the linamarin/lotaustralin ratio in the present flax microgreens was clearly higher. The diglycosylated species neolinustatin and linustatin were semi-quantified at much lower levels, namely 0.0897 ± 0.0014 and 0.79 ± 0.07 mg/g DW, respectively. These values are slightly lower than those reported by Zuk et al. [21] for flax plants harvested after 7 and 14 days, where both compounds ranged between 0.3 and 1.2 mg/g DW. It is worth noting that all quantified or semi-quantified CNGs were detected at concentrations above the LOD and LOQ values expressed in mg/g DW, calculated from those established from the calibration curve of the lotaustralin standard and corresponding to 0.0008 mg/g DW and 0.003 mg/g DW, respectively.

As discussed in the Introduction Section, current European regulations relevant to CNGs concern the amount of HCN that can potentially be released from food products and are generally expressed as mg/kg of product [14]. Therefore, the interpretation of CNG content in toxicological terms requires conversion into potential HCN release. Since enzymatic hydrolysis of a given CNG is expected to yield a sugar and a cyanohydrin molecule, which can further decompose to release HCN, a theoretical maximum HCN content can be estimated from the measured glycoside levels. This calculation represents a theoretical worst-case scenario and does not correspond to directly measured HCN release. Based on the CNG levels measured in flax microgreens, complete hydrolytic conversion would correspond to approximately 325 mg HCN/kg, well above the limit established for flaxseed marketed for direct consumption (150 mg/kg). However, complete conversion is unlikely under physiological or food-use conditions, because several factors, including enzyme activity, pH, and temperature [32], strongly affect hydrolysis efficiency and HCN release. Considering conversion factors of 1%, 10%, and 25% of the quantified CNGs into HCN, the estimated values in flax microgreens would be approximately 3, 33, and 81 mg/kg, respectively. These conversion values were adopted only as a pragmatic and conservative estimate for discussion purposes, since no direct hydrolysis experiments under simulated processing or gastrointestinal conditions were performed in the present study. The resulting values remain below the current EU limit for flaxseed, although they are comparable to or higher than values reported for other foods, such as chopped almonds/apricot kernels and cassava roots.

The quantitative results discussed so far have to be interpreted as representative of the specific vegetal material analyzed during this study; thus, considering that CNGs accumulation in flax is influenced by genotype and development, they cannot be broadly generalized to all flax microgreens. Nonetheless, the reported findings emphasize the importance of assessing CNG content in innovative vegetable products such as microgreens and suggest that dedicated safety considerations for this product category may be warranted. Future studies should therefore directly evaluate HCN release from flax microgreens under realistic consumption and digestion conditions, to refine the toxicological relevance of the present findings.

## 3. Materials and Methods

### 3.1. Chemicals

LC-MS grade water, methanol, acetonitrile, ammonium formate, sodium acetate, and formic acid were purchased from Merck KGaA (Darmstadt, Germany), as well as the analytical standards of dhurrin (≥95% (HPLC)) and lotaustralin (≥95% (LC/MS-ELSD)). Standard solutions for the mass spectrometer calibration were purchased from Thermo Scientific (Waltham, MA, USA).

### 3.2. Cultivation of Broccoli Raab, Kale and Flax Microgreens

*Brassica* microgreens were grown from 9 January to 9 March 2023, in a cold-frame greenhouse at the University of Bari Aldo Moro (41°06′ N, 16°52′ E, Bari, Southern Italy). Two local *Brassicaceae* genotypes were used: *Brassica rapa* L. subsp. *sylvestris* L. Janch. var. *esculenta* Hort, local variety ‘Cima di rapa novantina’ (broccoli raab); *Brassica oleracea* L. var. *acephala*, local variety ‘Cavolo riccio’ (kale). Cultivation was performed by sowing seeds at a density of 4 seeds/cm^2^. Daily irrigation was achieved using rainwater until germination; post-germination fertigation applied half-strength Hoagland nutrient solution twice daily. During the cultivation period, the natural photoperiod was 13/11 h (light/dark). The average day/night temperatures inside the greenhouse were 12 ± 2/8 ± 1 °C, and relative humidity was maintained at 60%. The half-strength Hoagland nutrient solution (NS) composition (mg·L^−1^) was as follows: 119 nitrogen, 16 phosphorus, 24 magnesium, 116 calcium, 58.4 potassium, 54 sulfur, 1.12 iron, 0.27 manganese, 0.13 zinc, 0.27 boron, 0.03 copper, and 0.01 molybdenum, resulting in an electrical conductivity (EC) of 1.8 dS·m^−1^ and a pH of 6.3. The nitrogen source was NO_3_^−^-N:NH_4_^+^-N at a ratio of 84:16. Microgreens were harvested at the first true-leaf stage (18 days for broccoli raab, 25 days for kale) and the fresh cut microgreens were immediately frozen in liquid nitrogen, and stored at −20 °C. Subsequently, the samples were freeze-dryed for 4 days in a ScanVac CoolSafe 55-9 Pro freeze-dryer (LaboGene ApS, Allerød, Denmark) and then stored at −18 °C until analyte extraction.

Seeds of flax (*Linum usitatissimum* L.) were purchased from Selex (Trezzano sul Naviglio, Italy) and adopted for the present study after their germinating ability was assessed in Petri dishes. The seeds were sown on a substrate consisting of a mixture of peat (50% white − 50% black peat mixture, Brill 3 Special, Brill Substrates, Georgsdorf, Germany) contained in a 150 cm^2^ cultivation plastic tray (with holes on the bottom), with a density of 3 seeds/cm^2^. The same cultivation conditions of *Brassica* species were adopted. After 10 days, once the development of seedlings and the appearance of the first true leaves were assessed, harvesting was carried out by cutting the microgreens just above the surface of the growing medium. The freshly cut flax microgreens were then processed like those of *Brassica* plants and stored under identical conditions until analyte extraction.

### 3.3. Standard Solutions and Extraction of Cyanogenic Glycosides from Microgreens

Standard stock solutions (1 mg/mL each) of dhurrin and lotaustralin were prepared in water and DMSO, respectively. A 50 mg/L working solution was prepared for each of the two compounds via dilution of the respective stock solution in water:methanol (80:20 *v*/*v*). The calibration for lotaustralin in pure solvent was obtained by analyzing standard solutions in a concentration range of 0.5–10 mg/L. Following dilution, the final DMSO content in the injected solutions was below 1%, and no adverse effects on chromatographic peak shape or ionization efficiency were observed. Limit of detection (LOD) and limit of quantification (LOQ) values were evaluated as three and ten times, respectively, the ratio between the intercept standard deviation and the slope of the calibration line.

The extraction of CNGs from lyophilized vegetable samples was performed by modifying a protocol already available in the literature [21]. In the present case, a pooled biological sample was obtained for each plant under study by collecting and subjecting to freeze-drying several microgreen seedlings grown under the same conditions, so that sufficient freeze-dried material was available for subsequent experiments. In a preliminary extraction experiment, 0.2 g of lyophilized product was suspended into 2 mL of solvent, corresponding to a methanol/water mixture of 70:30 (*v*/*v*). However, the solvent volume was unable to ensure complete sample dispersion. A 5 mL volume was thus adopted subsequently to suspend the 0.2 g amount of sample. Sonication was performed using an ultrasound bath at 25 °C, power 100%, and frequency 40 Hz for 15 min. The sample suspension was then centrifuged at 25 °C and 6000 rpm for 15 min, and the supernatant was collected. This procedure was based on previous experience on freeze-dried microgreen matrices and was subsequently repeated on the residual pellet; then, the two supernatants were pooled and dried using a SpeedVac™ SPD140DDA apparatus (Thermo Fisher Scientific, Waltham, MA, USA), operated at 35 °C and with a ramp pressure equal to 5. These steps of extraction were repeated four times during a specific experiment, aiming at evaluating the step-by-step recovery related to the extraction procedure. All dried samples were reconstituted in 1 mL of methanol/water 20:80 (*v*/*v*), and centrifuged at 25 °C, 14,500 rpm for 10 min before RPLC-ESI-HRMS analysis. The matrix effect was also evaluated in the case of flax microgreens by analyzing the diluted flax microgreens extract spiked with lotaustralin standard solution in the concentration range 0.5–10 mg/L.

### 3.4. RPLC-ESI-HRMS Instrumentation and Operating Conditions

Analyses by reversed-phase liquid chromatography coupled with high-resolution/accuracy mass spectrometry were conducted using an Ultimate 3000 UHPLC chromatographic system coupled with a quadrupole-Orbitrap mass spectrometer (Q-Exactive, Thermo Scientific, Waltham, MA, USA) equipped with a higher collisional-energy dissociation (HCD) cell and an electrospray (ESI) source (Thermo Scientific, Waltham, MA, USA), which was operated in negative or positive ion mode, according to the case. The chromatographic separations were conducted at a flow rate of 0.200 mL/min and at 25 °C using an Ascentis Express C18 column (150 × 2.1 mm ID, 2.7 μm particle size), equipped with an Ascentis Express C18 pre-column (5 × 2.1 mm ID) (Supelco, Merck KGaA, Darmstadt, Germany). Different chromatographic and mass spectrometric conditions were evaluated to find the best parameters for the separation and ionization of cyanogenic glycosides. Initially, experiments were performed using water and acetonitrile as solvents A and B, respectively, both containing 0.1% (*v*/*v*) formic acid, and with the ESI source operating in negative ion mode. The chromatographic separation was performed using the following gradient elution (GE #1, see also Figure S4) program: 0–5 min at 5% of solvent B; 5–8 min, linear increase from 5% to 15% of B; 8–25, min linear increase from 15% to 18% of B; 25–28 min, linear increase from 18% to 40% of B; 28–30 min, linear increase from 40% to 95% of B; 30–40 min, isocratic at 95% of B; 40–41 min, linear decrease from 95% to 5% of B; 41–55 min, re-equilibration step. A further chromatographic method employed a third solvent (C), consisting of water containing 20 mM ammonium formate, whose percentage in the mobile phase was maintained constant at 5% during the run, while the percentage of mobile phase A was adjusted so that the percentage of solvent B was the same, for a specific time, as that described for the previous program. During the exploratory runs based on the described gradient elution, MS spectra were acquired in both positive and negative polarity modes. A third chromatographic separation procedure was carried out using water containing 2 mM sodium acetate and 0.1% formic acid as mobile phase A, and acetonitrile with 0.1% formic acid as mobile phase B. The same gradient program described for the first method was applied.

The ESI and ion optics parameters were set as follows for MS acquisitions: sheath gas flow rate 35/40 in positive/negative polarity (arbitrary units), auxiliary gas flow rate 15/10 in positive/negative polarity (arbitrary units), spray voltage ±3.5 kV, capillary temperature 320 °C, S-lens radio frequency level 100 (arbitrary units). Full-MS spectra were acquired in the *m*/*z* range 100–1500 at a 70,000 resolving power, using an automatic gain control (AGC) target of 3 × 10^6^ counts/s and an ion injection time of 100 ms. The following conditions were adopted for tandem mass spectrometry (MS/MS) analyses: normalized collision energy (NCE) 35 (a.u.), resolving power 17,500, automatic gain control (AGC) 1 × 10^5^ counts/s, HCD cell fill time 50 ms, precursor ion isolation window width 1 *m*/*z*. The control of the LC-MS instrumentation and the first processing of data were performed by the Xcalibur software 2.2 SP1.48 (Thermo Scientific). The final visualization of mass spectra and chromatograms was performed by SigmaPlot 14.0.

## 4. Conclusions

RPLC-ESI(+)-HRMS, optimized using lotaustralin and dhurrin as model compounds, proved to be a sensitive approach for determining cyanogenic glycosides in microgreens by exploiting stable sodium adduct formation. The corresponding HRMS/MS spectra provided diagnostic product ions for reliable CNG recognition in complex matrices. A clear compositional difference emerged among the investigated species: no detectable CNGs were found in kale and broccoli raab microgreens, whereas flax microgreens contained several CNGs, consistent with previous reports for early flax growth stages. Linamarin and lotaustralin were the predominant compounds, while the diglycosylated species were detected as multiple peaks consistent with isomeric forms of linustatin and neolinustatin. The concentrations estimated in flax microgreens indicate a relevant cyanogenic potential, with calculated HCN release values, assuming 1%, 10% and 25% conversion rates, ranging between 3 and 81 mg/kg, thus approaching toxicological and regulatory concerns. These findings should not be generalized to other flax microgreens or related plant materials, whose composition may vary depending on genotype and developmental stage. Nonetheless, they show that microgreens should be assessed not only for nutritional value but also for toxicologically relevant secondary metabolites. Flax microgreens deserve targeted monitoring for CNG occurrence. However, actual HCN release upon consumption still needs experimental verification, and the lack of authentic standards for all analytes limited full quantification of some compounds. Overall, the proposed method can be considered a useful tool for food safety assessment and for future studies on CNG variability across plant species, growth stages, and cultivation conditions.

## Data Availability

The raw data supporting the conclusions of this article will be made available by the authors on request.

## Supplementary Materials

The following supporting information can be downloaded at https://www.mdpi.com/article/10.3390/plants15091358/s1. Figure S1. General chemical structure of cyanogenic glycosides (CNGs), showing glucose as the sugar moiety. R1 and R2 denote variable substituents that differ among individual compounds; Figure S2. Proposed fragmentation pathways accounting for the formation of the product ions detected in the ESI(+)-HCD-MS/MS spectra of the sodium adducts [M+Na]^+^ of the lotaustralin (*m*/*z* 284.1) and dhurrin (*m*/*z* 334.1); Figure S3. Proposed fragmentation pathways accounting for the formation of the compound-specific product ions with *m*/*z* values below 100 detected in the ESI(+)-HCD-MS/MS spectra of the sodium adducts of lotaustralin (*m*/*z* 284.111) and linamarin (*m*/*z* 270.095) identified in flax microgreens; Figure S4. Total ion current (TIC) chromatogram of flax microgreen extract obtained by RPLC-ESI-HRMS using the gradient elution program adopted in this study (GE #1, blue line). For less complex matrices, a shorter gradient elution program (GE #2, red line) may be used as a suitable alternative (see the main text for details). The GE #2 program was as follows: 0–5 min, 5% B (isocratic); 5–8 min, 5–15% B (linear); 8–15 min, 15–16.5% B (linear); 15–16 min, 16.5–95% B (linear); 16–26 min, 95% B (isocratic); 26–27 min, return to the initial composition; followed by 10 min of re-equilibration; Figure S5. Proposed fragmentation pathways accounting for the formation of the product ions detected in the ESI(+)-HCD-MS/MS spectra of the sodium adducts of the isomeric linustatin- and neolinustatin-related species detected in flax microgreens; Figure S6. Calibration plots obtained for lotaustralin using (a) standard solutions and (b) spiked flax microgreens extract aliquots. Data were acquired over the concentration range 0.5–10 mg/L (n = 3 per level). The plots show the EIC peak area of the sodium adduct ([M+Na]^+^, *m*/*z* 284.111) as a function of analyte concentration. Linear regression parameters are as follows: (a) intercept = (−9 ± 2) × 10^6^, slope = (439 ± 5) × 10^5^; (b) intercept = (19 ± 2) × 10^6^, slope = (456 ± 4) × 10^5^; Table S1: Cyanogenic glycosides identified in flax microgreens. The table reports monoisotopic *m*/*z* values for the [M+Na]^+^ precursor ions and for diagnostic product ions detected in the corresponding MS/MS spectra, identification levels, and concentrations expressed as mg/g of dry weight (DW). Mean values and standard deviations (n = 3) are reported for concentrations.
