# Supplementary material for: Liquid Chromatography coupled with Mass Spectrometry as an Analytical Strategy to Assess the Occurrence of Potentially Toxic Cyanogenic Glycosides in Edible Microgreens
Source: Plants (Basel). 2026 Apr 29;15(9):1358. doi: 10.3390/plants15091358 (PMC13164923; doi:10.3390/plants15091358)
Supplement: Supplementary file 1 [file plants-15-01358-s001.zip › plants-4238777-supplementary.pdf]

Supplementary Material

LC-ESI-HRMS as an analytical strategy to assess the occurrence of potentially toxic cyanogenic glycosides in edible microgreens

Mariachiara Bianco[1,2,*], Ilario Losito[1,2,*], Beniamino Leoni[3], Onofrio Davide Palmitessa[3], Massimiliano Renna[3], Pietro Santamaria[2,3], Cosima Damiana Calvano[1,2], Tommaso R.I. Cataldi[1,2]

[1]*Dipartimento di Chimica e* [2]*Centro interdipartimentale SMART, Università degli Studi di Bari Aldo Moro, via E. Orabona 4, 70126, Bari, Italy;* [3]*Dipartimento di Scienze del Suolo, della Pianta e degli Alimenti - Università degli Studi di Bari Aldo Moro, via Orabona 4, 70126, Bari, Italy*

Number of Figures: 6
Number of Table: 1

***Author for correspondence**; email: mariachiara.bianco@uniba.it, ilario.losito@uniba.it

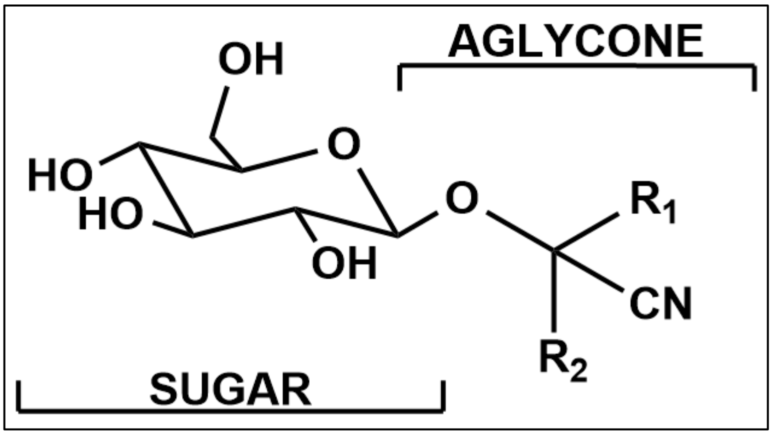

Figure S1. General chemical structure of cyanogenic glycosides (CNGs), showing glucose as the sugar moiety. R_1 and R_2 denote variable substituents that differ among individual compounds.

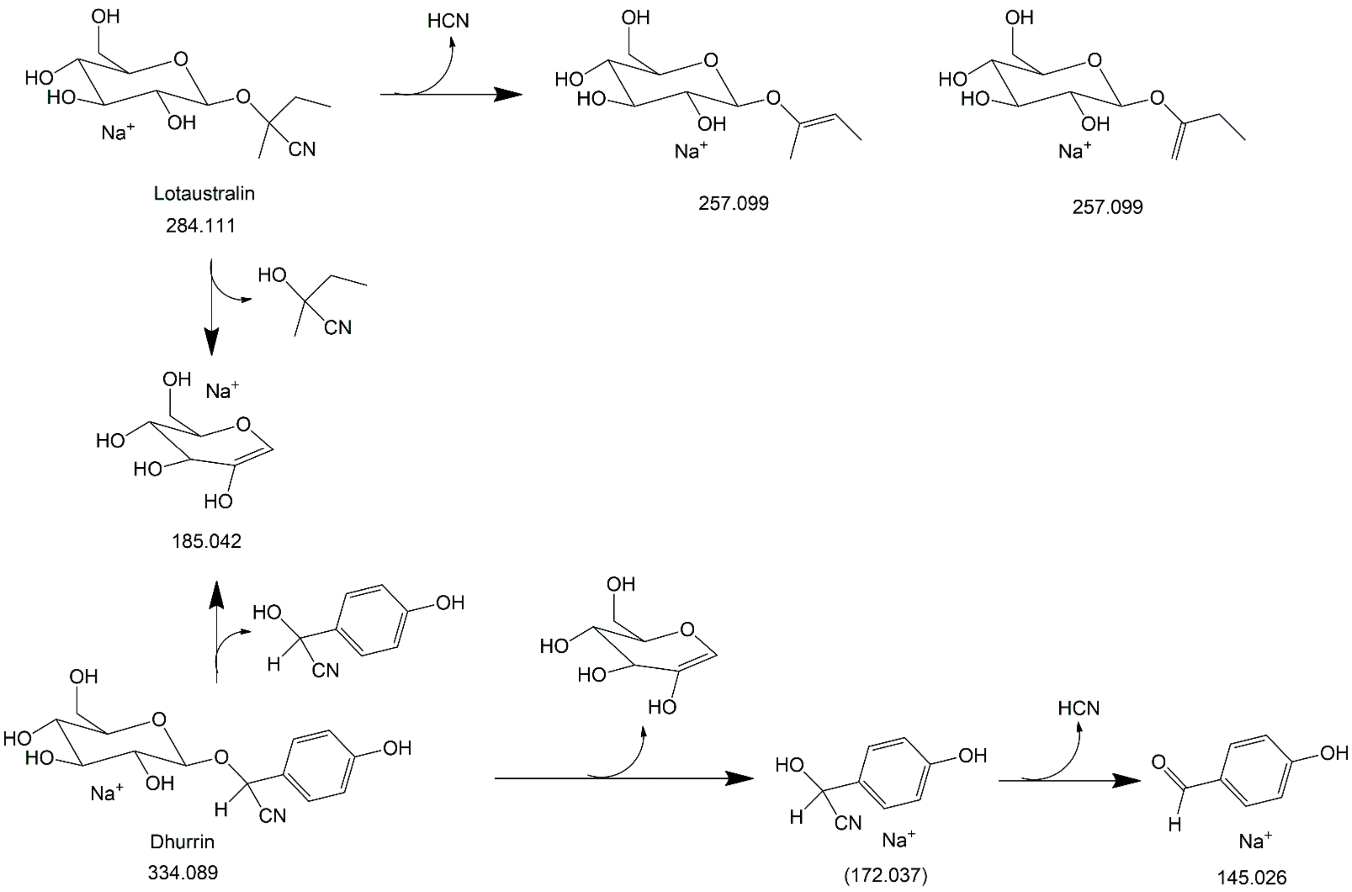

Figure S2. Proposed fragmentation pathways accounting for the formation of the product ions detected in the ESI(+)-HCD-MS/MS spectra of the sodium adducts $[M+Na]^+$ of lotaustralin (*m/z* 284.1) and dhurrin (*m/z* 334.1). Under HCD conditions, fragmentation of both precursors is initiated by a glycosidic bond cleavage, with neutral loss of a cyanohydrin. For dhurrin and lotaustralin, this process yields the ion at *m/z* 185.042, assigned to the sodium adduct of dehydrated glucose, $[\text{glucose} - H_2O + Na]^+$. A second dhurrin-specific fragment, at *m/z* 145.026, is consistent with the consecutive losses of dehydrated glucose (Δ = 162 Da) and HCN (Δ = 27 Da), with the latter being particularly favoured, as no significant peak signal was observed in the MS/MS spectrum for the *m/z* 172.037 ion, resulting from the dehydrated glucose loss. For lotaustralin, the characteristic ion at *m/z* 257.099 corresponds to HCN loss (Δ = 27 Da) from the sodium adduct precursor. Two alternative isomeric structures are proposed here for this ion, formed through competing fragmentation pathways of the α-hydroxynitrile moiety under HCD activation.

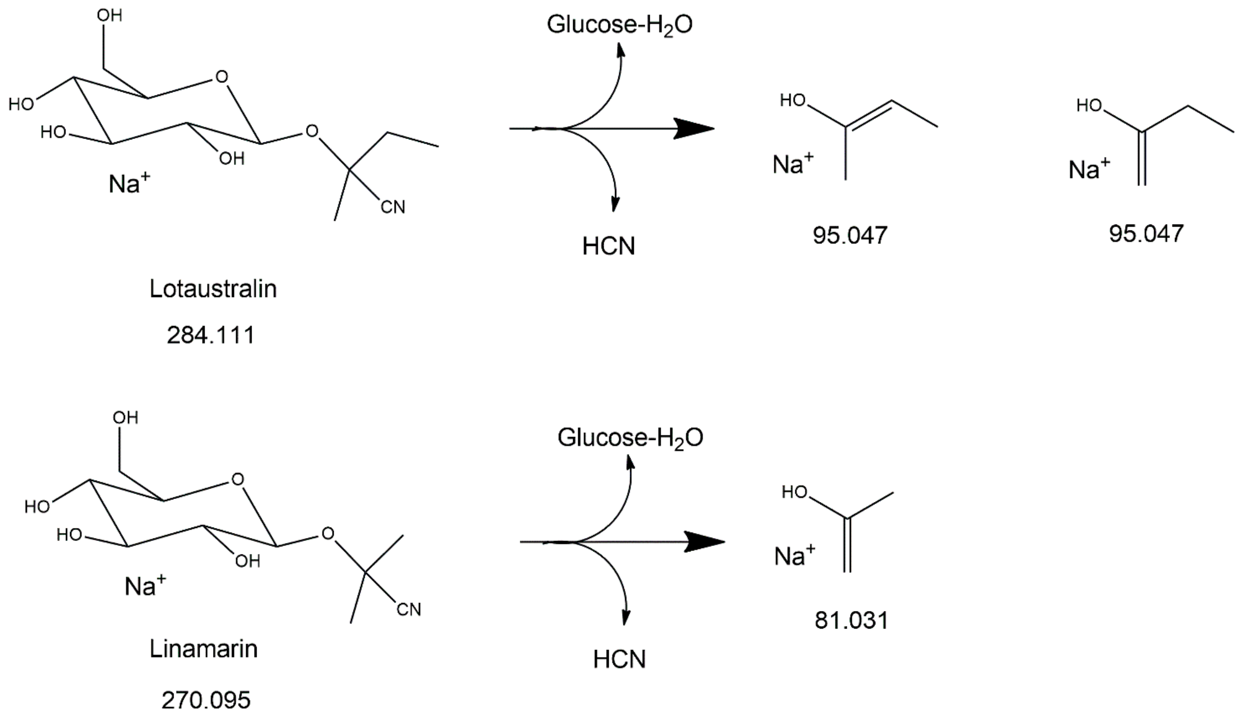

Figure S3. Proposed fragmentation pathways accounting for the formation of the compound-specific product ions with *m/z* values below 100 detected in the ESI(+)-HCD-MS/MS spectra of the sodium adducts of lotaustralin (*m/z* 284.111) and linamarin (*m/z* 270.095) identified in flax microgreens. Under HCD conditions, the precursor ions undergo glycosidic bond cleavage, leading to the neutral loss of a dehydrated glucose moiety (glucose − H_2O, Δ = 162 Da). This process generates an internally excited aglycone, which undergoes rapid decomposition involving the elimination of hydrogen cyanide (HCN, Δ = 27 Da), according to two possible pathways in the case of lotaustralin and a single pathway in the case of linamarin. For both linamarin and lotaustralin, this fragmentation leads to the formation of diagnostic ions in the low-mass region (*m/z* 81.031 and 95.047, respectively); in the case of lotaustralin, two possible structures can be proposed for the resulting product ion.

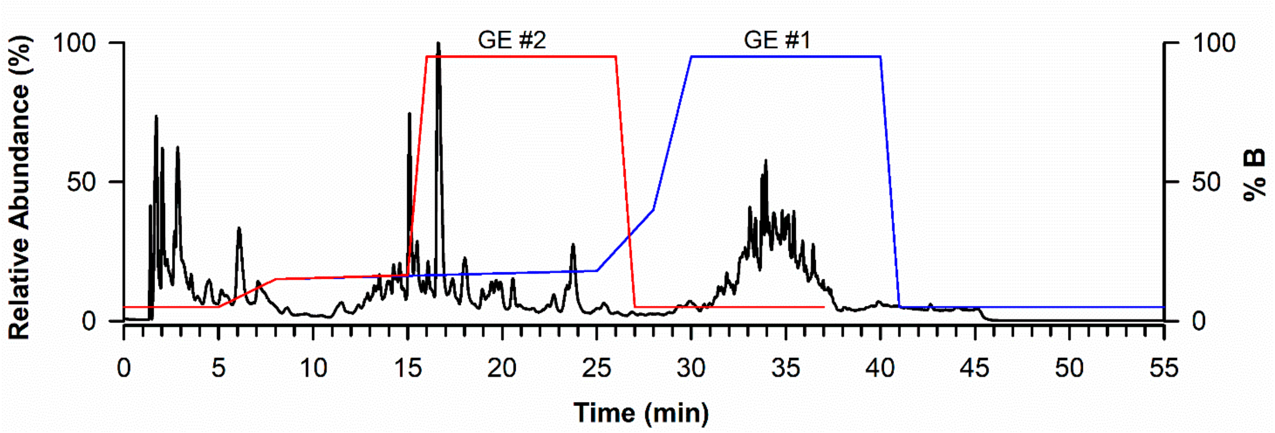

Figure S4. Total Ion Current (TIC) chromatogram of a flax microgreen extract obtained by RPLC-ESI-HRMS using the gradient elution program adopted in this study (GE #1, blue line). For less complex matrices, a shorter gradient elution program (GE #2, red line) may represent a suitable alternative (see the main text for details). The GE #2 program was as follows: 0–5 min, 5% B (isocratic); 5–8 min, 5–15% B (linear); 8–15 min, 15–16.5% B (linear); 15–16 min, 16.5–95% B (linear); 16–26 min, 95% B (isocratic); 26–27 min, return to the initial composition; followed by 10 min of re-equilibration.

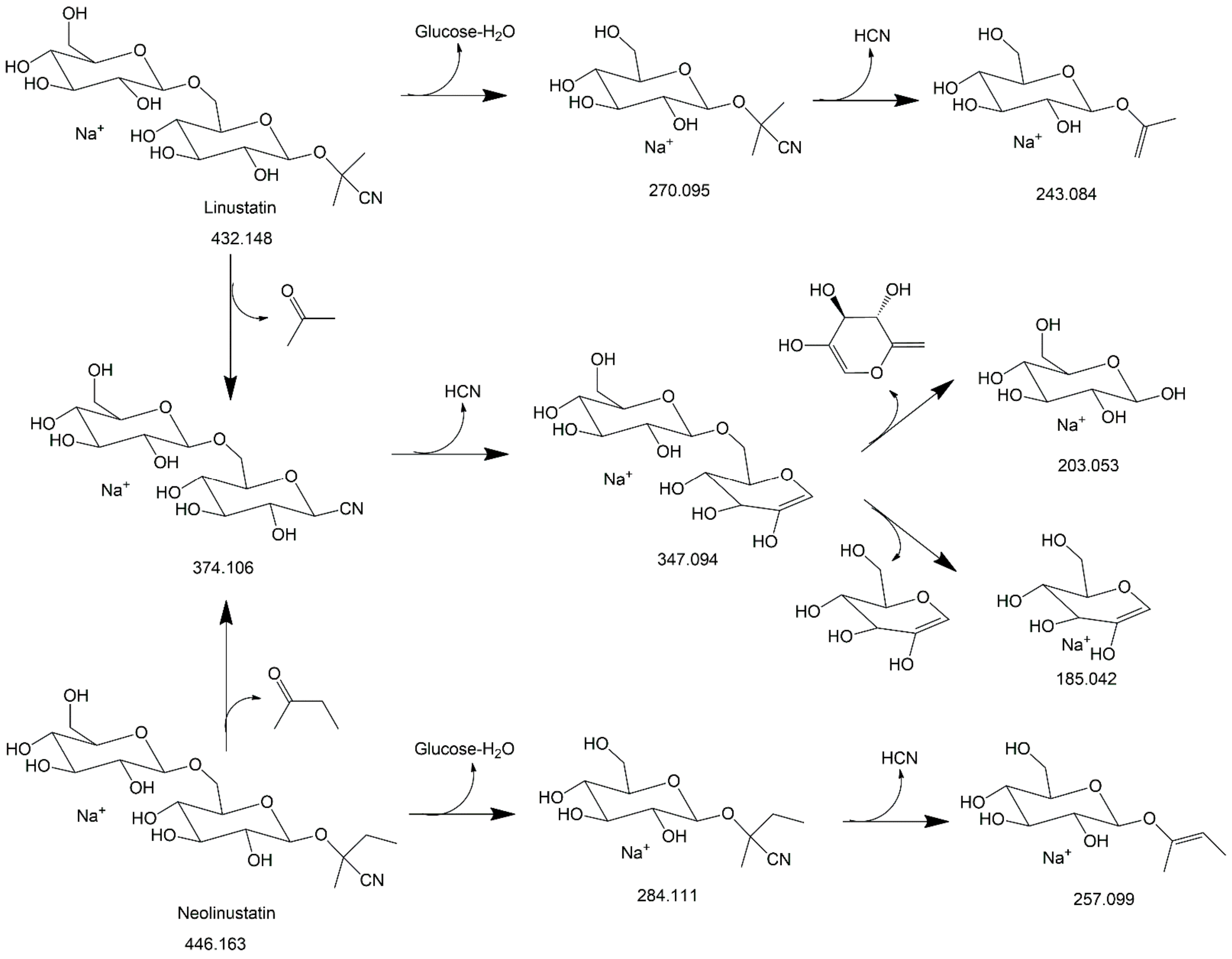

Figure S5. Proposed fragmentation pathways accounting for the formation of the product ions detected in the ESI(+)-HCD-MS/MS spectra of the sodium adducts of the isomeric linustatin- and neolinustatin-related species detected in flax microgreens. Under HCD conditions, linustatin yields a *m/z* 270.095 product ion after loss of dehydrated glucose (Δ = 162 Da), followed by HCN loss (Δ = 27 Da) to form the *m/z* 243.084 ion. An alternative pathway gives a *m/z* 374.106 ion, which further fragments to *m/z* 347.094 and then to the sugar-derived ions at *m/z* 203.053 and 185.042. Neolinustatin undergoes an analogous loss of dehydrated glucose (Δ = 162 Da) to give a *m/z* 284.111 ion, followed by HCN elimination (Δ = 27 Da) to yield a *m/z* 257.099 ion. The latter ion can be interpreted in terms of two alternative isomeric structures, yet only the presumably more stable one, including a more substituted alkenic moiety, is reported in the figure, whereas the alternative one features a methylidene moiety.

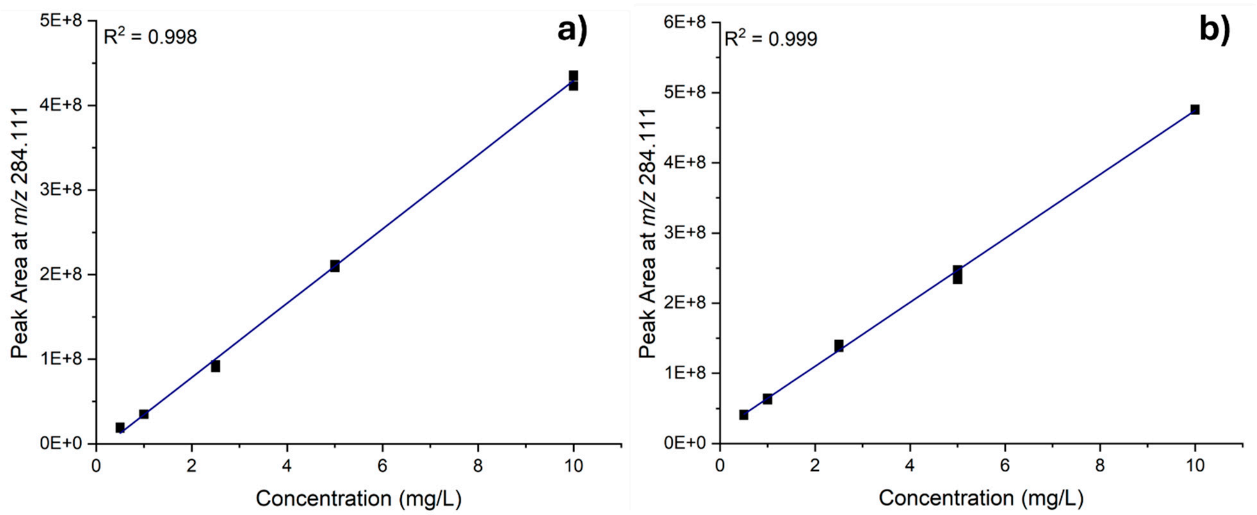

Figure S6. Calibration plots obtained for lotaustralin using: (a) standard solutions and (b) spiked flax microgreens extract aliquots. Data were acquired over the concentration range 0.5–10 mg/L (n = 3 per level). The plots show the EIC peak area of the sodium adduct ($[M+Na]^+$, *m/z* 284.111) as a function of analyte concentration. Linear regression parameters are: (a) intercept = $(-9 \pm 2) \times 10^6$, slope = $(439 \pm 5) \times 10^5$; (b) intercept = $(19 \pm 2) \times 10^6$, slope = $(456 \pm 4) \times 10^5$.

Table S1. Cyanogenic glycosides identified in flax microgreens. The table reports monoisotopic *m/z* values for the $[M+Na]^+$ precursor ions and for diagnostic product ions detected in the corresponding MS/MS spectra, identification levels, and concentrations expressed as mg/g of dry weight (DW). Mean values and standard deviations (n = 3) are reported for concentrations.

CNGs	$[M+Na]^+$ as precursor ion (*m/z*)	Diagnostic product ions (*m/z*)	Identification level [a]	Quantification in flax microgreens (mg/g DW)
Linamarin	270.095	243.085; 185.043; 81.032	2	24 ± 3*
Lotaustralin	284.111	257.099; 185.043; 95.047	1	5.5 ± 0.6
Linustatin	432.148	405.136; 374.105; 347.094; 270.095; 243.084; 203.052; 185.043	3	0.79 ± 0.07*
Neolinustatin	446.163	419.152; 374.105; 347.094; 284.111; 257.099; 203.052; 185.043	3	0.0897 ± 0.0014*

[a] Identification levels were assigned according to Schymanski et al. [29]: Level 1 (confirmed structure by reference standard); Level 2 (probable structure by library match or diagnostic evidence); Level 3 (tentative candidate). *Values obtained through semi-quantification (referring to the most structurally similar available standard).
